# Blastomycosis of the psoas muscles

**DOI:** 10.1016/j.idcr.2021.e01156

**Published:** 2021-05-09

**Authors:** Giuseppe Ietto, Andreina Baj, Cristiano Parise, Elia Zani, Domenico Iovino, Giulio Carcano, Daniela Dalla Gasperina

**Affiliations:** a Emergency and Transplant Surgery Department, ASST-Settelaghi and University of Insubria, Varese, Italy; b Department of Medicine and Surgery, University of Insubria, Varese, Italy; c Infectious Disease Department, ASST-Settelaghi and University of Insubria, Varese, Italy

**Keywords:** Blastomycosis, Abscess, Psoas muscles, Itraconazole

A 27 year old African man, who arrived in Italy from Senegal four months before, was admitted after several weeks of low-grade fever and mild pain, mainly localized in the inferior left abdominal quadrant and at the root of the thighs bilaterally. A preceding disseminated tuberculosis has been reported but no concurrent conditions of immunosuppression nor HIV infection were diagnosed. The patient seemed severely suffering and cachectic. CT-scan noticed multiple large and confluent fluid collections deeply in both psoas muscles (up to 9 cm), associated with multiple bone lytic lesions of the pelvis Figures 1, 2, and 3. The clinical conditions got worse, despite the broad-spectrum antibacterials (rifampicin, doxycycline and ciprofloxacin combined therapy). Percutaneous drainage was unsuccessful because the fluid was too thick. Therefore, the collections were drained sequentially through a retroperitoneal surgical approach. The fluid samples tested positive for *Blastomyces dermatitidis,* through molecular amplification and sequencing of the 18S region. Oral itraconazole (200 mg 3 times/day for 3 days, and then 200 mg bid) therapy prolonged for six months led to progressive clinical improvement, until complete recovery. Blastomycosis is a rare fungal infection mainly responsible for pulmonary disease. Less than half of symptomatic cases develop an extrapulmonary infection. Cultural recognition is needed for diagnosis and specific treatment. The patient agreed to publish this case and a written informed consent was obtained and available from the corresponding author.

**Fig. 1:**
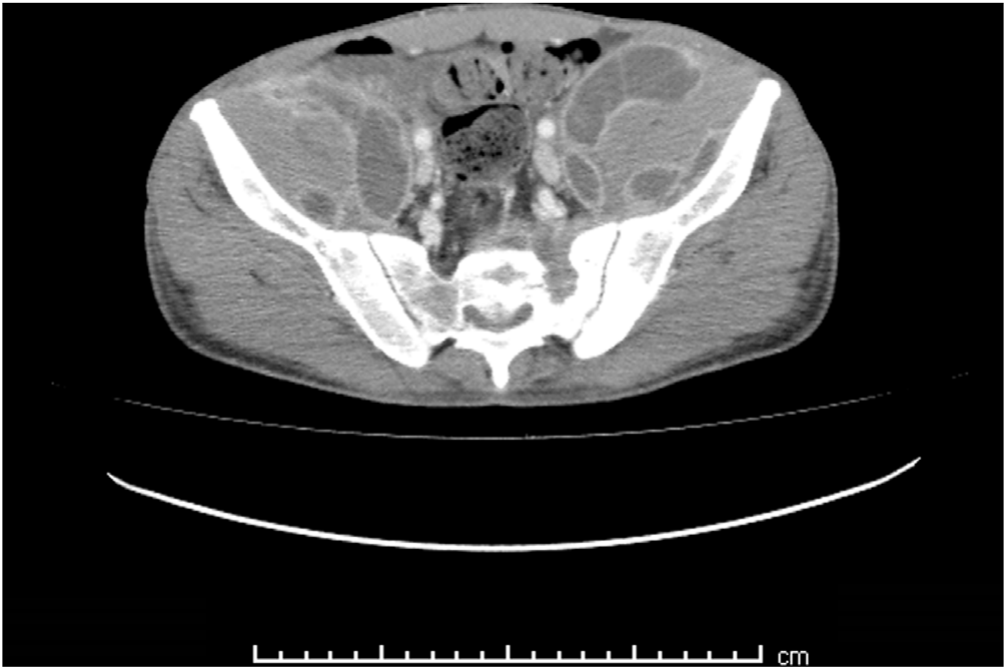
CT-scan axial view: large abscesses in both psoas muscles and multiple bone lytic lesions of the pelvis.

**Fig. 2:**
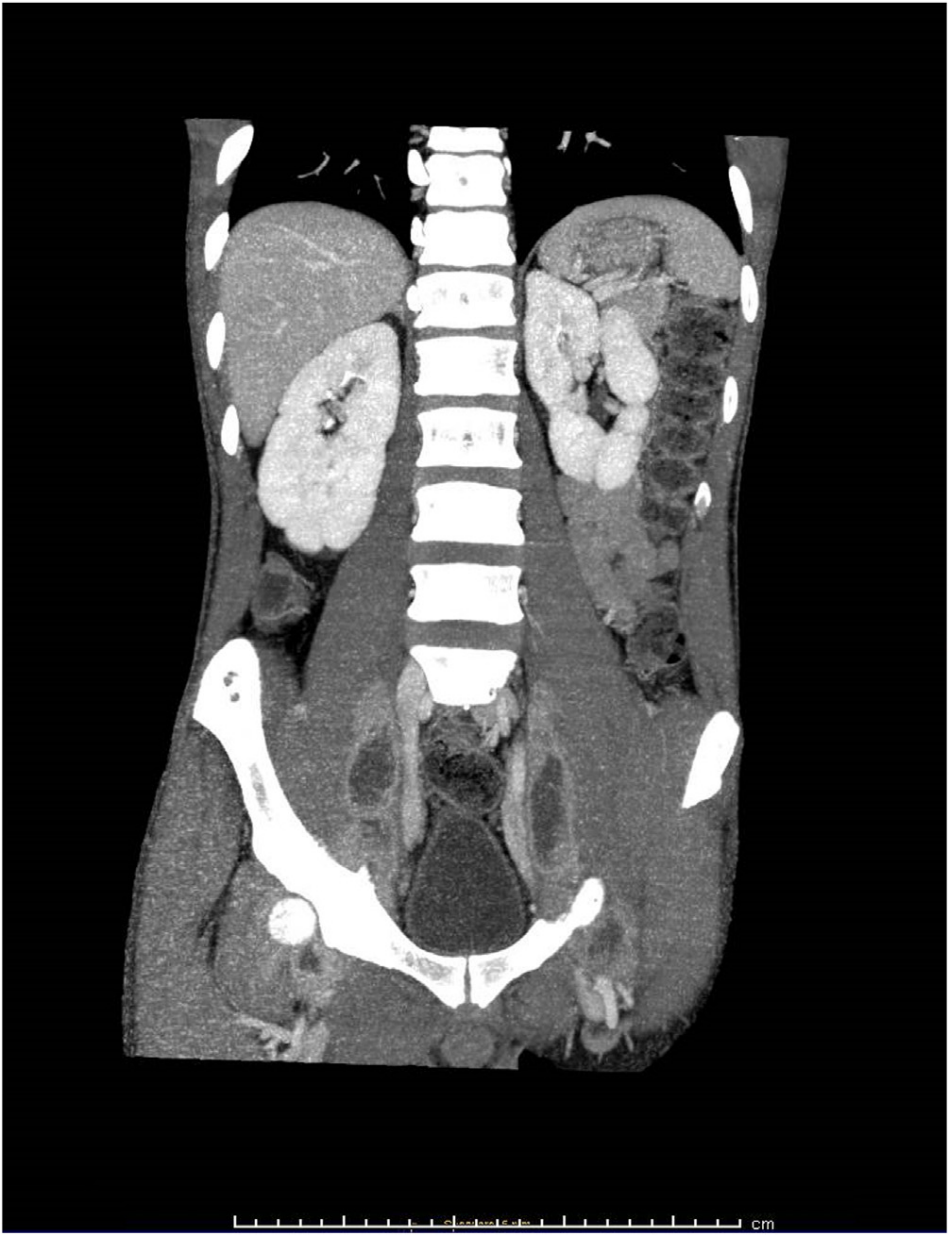
CT-scan coronal view: the abscesses were deeply in both the psoas muscles.

**Fig. 3:**
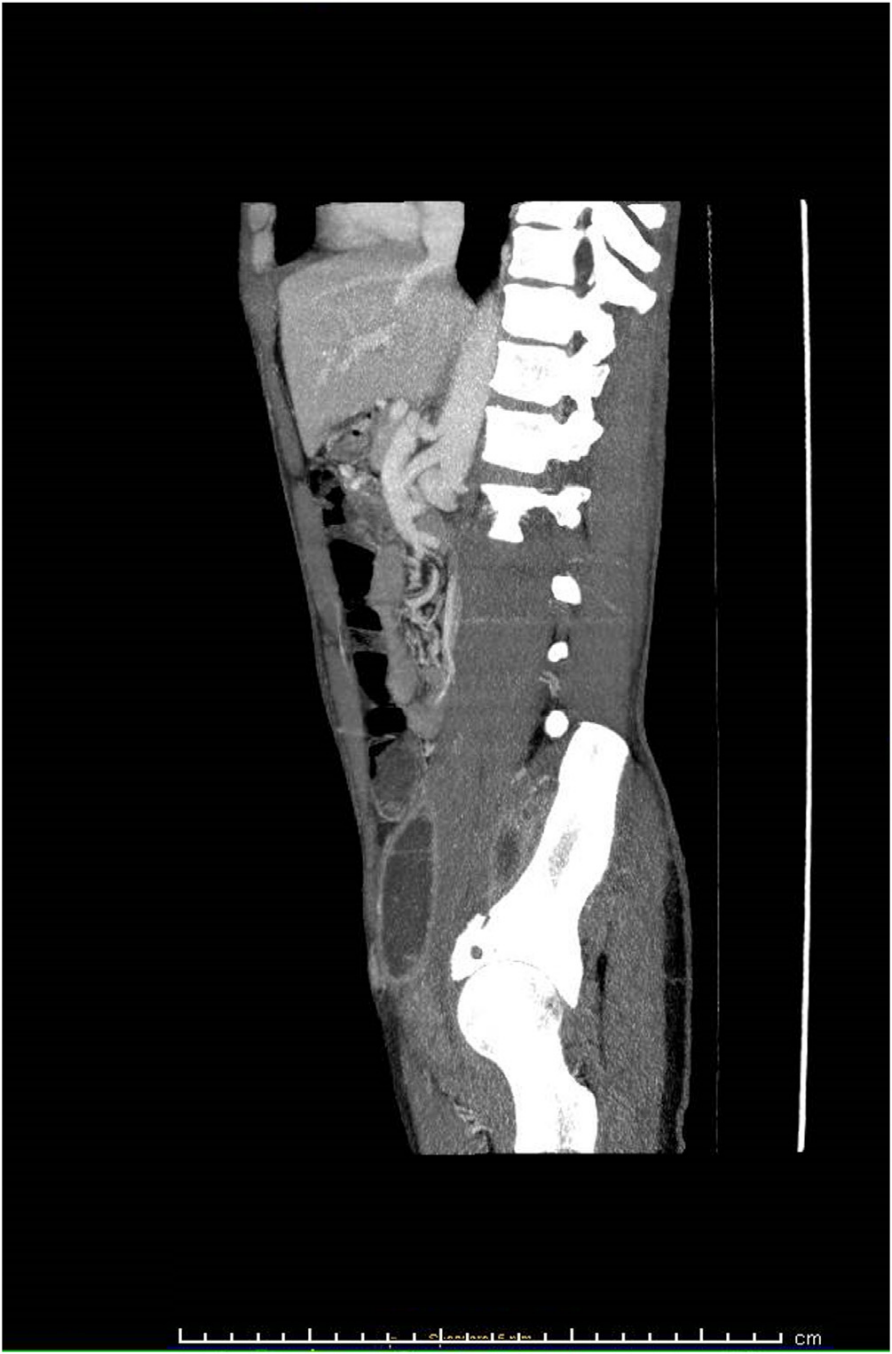
CT-scan sagittal view: the abscesses extended cranio-caudally up to 9 cm in length.

## Conflicts of interest

All authors declare no conflict of interest

## Sources of funding

No founding has been acquired for the present work

## Consent

Written informed consent was obtained from the patient for publication of this case report and accompanying images. A copy of the written consent is available for review by the Editor-in-Chief of this journal on request

## Ethical approval

None.

## Author contribution

First investigator, performed surgery, planned and wrote the paper.

Dr. Giuseppe Ietto performed surgery, planned and wrote the paper.

Dr.ssa Andreina Baj contributed to perform microbiological analysis and to write the paper.

Dr. Crisiano parise and Dr. Elia Zani contributed to data collection.

Dr. Domenico Iovino contributed to perform surgery and data collection.

Prof. Giulio Carcano revised the manuscript and supervised

